# Stratification according to recursive partitioning analysis predicts outcome in newly diagnosed glioblastomas

**DOI:** 10.18632/oncotarget.17322

**Published:** 2017-04-21

**Authors:** Fan Yang, Pei Yang, Chuanbao Zhang, Yongzhi Wang, Wei Zhang, Huimin Hu, Zhiliang Wang, Xiaoguang Qiu, Tao Jiang

**Affiliations:** ^1^ Department of Molecular Pathology, Beijing Neurosurgical Institute, Capital Medical University, Beijing, China; ^2^ Department of Neurosurgery, Beijing Tiantan Hospital, Capital Medical University, Beijing, China; ^3^ Department of Radiation Therapy, Beijing Tiantan Hospital, Capital Medical University, Beijing, China; ^4^ China National Clinical Research Center for Neurological Diseases, Beijing, China; ^5^ Center of Brain Tumor, Beijing Institute for Brain Disorders, Beijing, China

**Keywords:** glioblastoma, prognosis, recursive partitioning analysis, molecular marker, MGMT

## Abstract

Glioblastoma accounts for more than half of diffuse gliomas. The prognosis of patients with glioblastoma remains poor despite comprehensive and intensive treatments. Furthermore, the clinical significance of molecular parameters and routinely available clinical variables for the prognosis prediction of glioblastomas remains limited. The authors describe a novel model may help in prognosis prediction and clinical management of glioblastoma patients. We performed a recursive partitioning analysis to generate three independent prognostic classes of 103 glioblastomas patients from TCGA dataset. Class I (MGMT promoter methylated, age <58), class II (MGMT promoter methylation, age ≥58; MGMT promoter unmethylation, age <54, KPS ≥70; MGMT promoter unmethylation, age >59, KPS ≥70), class III (MGMT promoter unmethylation, age 54-58, KPS ≥70; MGMT promoter unmethylation, KPS <70). Age, KPS and MGMT promoter methylation were the most significant prognostic factors for overall survival. The results were validated in CGGA dataset.

This was the first study to combine various molecular parameters and clinical factors into recursive partitioning analysis to predict the prognosis of patients with glioblastomas. We included MGMT promoter methylation in our study, which could give better suggestion to patients for their chemotherapy. This clinical study will serve as the backbone for the future incorporation of molecular prognostic markers currently in development. Thus, our recursive partitioning analysis model for glioblastomas may aid in clinical prognosis evaluation.

## INTRODUCTION

Glioblastoma (GBM) accounts for the majority of diffuse gliomas in adults [1]. Despite intense efforts over the past several decades, the prognosis of patients with malignant glioma, particularly glioblastoma, remains dismal [2]. The median overall survival (OS) of patients with GBM is approximately 15–17 months with the current gold-standard first-line treatment, which is maximal safe resection and combination of radiotherapy with temozolomide chemotherapy [1–4]. The combination of molecular markers as directive signatures with radiotherapy and chemotherapy could be a promising treatment approach for patients with GBM.

Recently, numerous molecular biomarkers correlated with GBM have been discovered (for example. IDH1/2, ATRX, 1p19q codeletion, TERT and MGMT promoter methylation), which were found to be highly associated with patient prognosis and glioma heterogeneity. For instance, MGMT promoter methylation blocks MGMT protein expression and is predictive of chemotherapy sensitivity, specifically to alkylating agents such as temozolomide in patients with GBM [5, 6]. However, other clinical variables, such as age, Karnofsky performance scale (KPS), radiotherapy, and chemotherapy, also carry prognostic significance. Thus, further investigation is needed to determine how to weight the relative importance of molecular parameters and clinical factors and incorporate them into prognostication and treatment decisions.

Recursive partitioning analysis (RPA) enables classification of patients into successively more homogeneous prognostic groups based on multiple input variables [7]. This model can be applied to estimate prognosis and to help refine inclusion criteria for therapeutic trials. In this study, we performed RPA using routinely available clinical variables and molecular markers from The Cancer Genome Atlas (TCGA) to generate prognostic groups. The results were also validated in the independent CGGA dataset. Prognostic models have been generated in other brain tumors, including GBM [8–10], anaplastic astrocytoma [9], low-grade glioma [11], anaplastic oligodendroglioma [12], and primary central nervous system lymphoma [13]. However, this is the first report of an RPA model including both clinical and molecular factors for prognosis evaluation of patients with GBM. All the patients after the operation were treated with standard chemoradiation in order that this study can investigate more deeply the impact of molecular markers on patients with GBM. Our RPA model may be a useful prognostic tool for patients with GBM.

## RESULTS

### Patient, tumor, and treatment characteristics

In this study, 103 patients with GBM were enrolled from TCGA as training set and 116 GBM patients from CGGA were constituted the validation set (Table 1). TCGA cohort included 66 males and 37 females with a median age of 59 years (range, 21–83 years) and a median preoperative KPS of 70. Six molecular markers associated with prognosis were also shown in Table 1. In the CGGA dataset, there were 77(66%) men and median age was 49 years. Median preoperative KPS was 80 (Supplementary Table 1). The selection criteria of patients were that: (a) adult patients with primary GBM, patients younger than 18 years old and secondary and recur GBM were excluded; (b) patients were treated with standard chemoradiation and chemotherapy followed by adjuvant chemotherapy [15]; (c) we excluded patients with an OS time (defined as the interval from the date of diagnosis until death or the last follow-up) of < 30 days, since in these cases, death may have occurred due to factors other than GBM.

**Table 1:** Clinical and molecular pathology features between patients from TCGA and CGGA

Variable	TCGA (n=103)		CGGA (n=116)	
	No. of patients	Median OS (days)	No. of patients	Median OS (days)
**Gender**				
**Men**	66	397	77	520
**Women**	37	498	38	733
**Age**				
**<59**	49	476	95	733
**≥59**	54	372	21	520
**Preoperative KPS score**				
**<70**	24	357	23	487
**≥70**	79	640	69	681
**Radiotherapy**				
**Yes**	103	478	116	657
**No**	0		0	
**Chemotherapy**				
**Yes**	103	478	116	657
**No**	0		0	
**ATRX mutation**				
**Mutation**	8	439	/	/
**Wild type**	95	507	/	/
**TERT mRNA expression**				
**High**	50	387	20	563
**Low**	10	571	20	681
**NA**	43		76	
**TERT promoter mutation**				
**mutation**	13	446	10	
**Wild type**	2	414	21	965
**NA**	88		85	584
**1p19q codeletion**				
**Non-codeletion**	98	432	115	657
**Codeletion**	0		1	372
**NA**	5	458	0	
**IDH mutation**				
**Mutation**	9	498	18	1262
**Wild type**	94	446	98	563
**MGMT promoter methylation**				
**Methylation**	44	809	43	970
**Unmethylation**	59	369	73	455

OS: overall survival; / : CGGA dataset not supplied; NA: not available.

### Prognostic factors

After univariate analysis, multivariate Cox models were applied (Table 2). From the univariate analysis in TCGA, MGMT promoter methylation status was found to be significant and favorable prognostic factors (p < 0.01). Age (median age was 59 at GBM diagnosis) (p =0.04), KPS (median KPS was 70) (p = 0.05) were also statistically significant factors based on univariate analysis. In the multivariate model, KPS (HR 0.46, 95% CI 0.23-0.92, p =0.03) and MGMT promoter methylation status (HR 0.35, 95% CI 0.18-0.68, p <0.01) were independently associated with improved OS. It is well-known that chemotherapy and radiation are prognostic factors for GBM patients. The results of univariate and multivariate analysis for validation set were shown in Supplementary Table 1.

**Table 2:** Univariate and multivariate analysis of 103 patients with GBM in TCGA

Variable	Total (n=103)				
	No. of patients	P 1	HR	95%CI	P 2
**Gender**					
**Men**	66	0.16			
**Women**	37				
**Age**					
**<59**	49	0.04	1.22	0.68-2.20	0.51
**≥59**	54				
**Preoperative KPS score**					
**<70**	24	0.05	0.46	0.23-0.92	0.03
**≥70**	79				
**ATRX mutation**					
**Mutation**	8	0.46			
**Unmethylation**	95				
**TERT mRNA expression**					
**High**	50	0.07			
**Low**	10				
**NA**	43				
**TERT promoter mutation**					
**Mutation**	13	0.71			
**Wild type**	2				
**NA**	88				
**IDH mutation**					
**Mutation**	9	0.10			
**Wild type**	94				
**MGMT promoter methylation**					
**Methylation**	44	<0.01	0.35	0.18-0.68	<0.01
**Unmethylation**	59				

OS: overall survival; HR: Hazard ratio; NA: not available. P 1: the P value of univariate analysis; P 2: the P value of multivariate analysis.

### Recursive partitioning analysis

**Figure 1:**
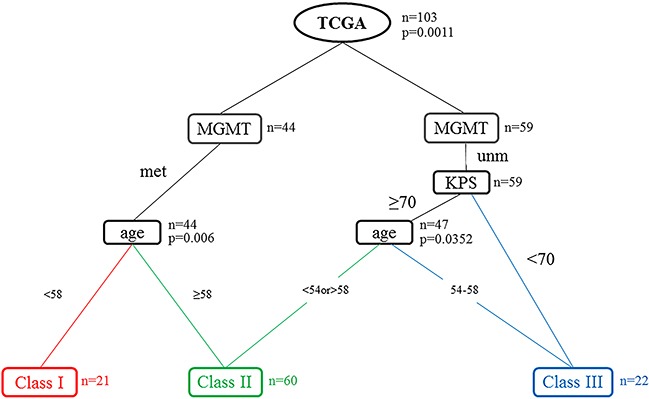
Recursive partitioning analysis (RPA) tree for the 103 patients in the TCGA data set The tree was evaluated as potential split points. The final decision tree is shown with terminal nodes and consolidation into three distinct prognostic classes using commonly available clinical variables. Abbreviations: KPS = Karnofsky performance scale; MGMT = MGMT promoter methylation; Met: Methylation; Unm: Unmethylation; n=the number of patients in the node; p=p-value.

We utilized RPA to predict OS with our dataset collected retrospectively from the TCGA. The optimal tree size was one with three leaves (Figure 1). Only three variables were included in the final model: age, KPS and MGMT promoter methylation status. We identified a primary split corresponding to MGMT promoter methylation status and secondary splits corresponding to age and KPS. Among patients with MGMT promoter methylation and age <58 were the strongest prognostic factors, and these patients survived longest. Furthermore, MGMT promoter methylation was the only prognostic factor in patients with age > 58. For patients with MGMT promoter unmethylation and KPS≥70, younger patients (age <54) exhibited increased OS compared to older patients (age >59). Finally, the worst prognosis was observed for patients with MGMT promoter unmethylation, KPS ≥70 and age band at 54-58, or patients with MGMT promoter unmethylation, KPS <70.

**Figure 2:**
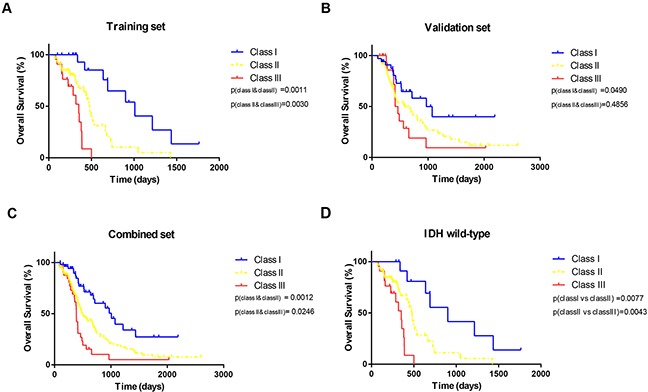
Kaplan-Meier curves The overall survival split according to subgroups derived from RPA for **(A)** training set **(B)** validation set, **(C)** combined set and **(D)** GBM, IDH-wildtype.

Therefore, RPA identified three distinct risk groups based on median survival similarity (Table 3): class I (MGMT promoter methylation, age <58), class II (MGMT promoter methylation, age ≥58; MGMT promoter unmethylation, age <54, KPS ≥70; MGMT promoter unmethylation, age >59, KPS≥70), class III (MGMT promoter unmethylation, age 54-58, KPS ≥70; MGMT promoter unmethylation, KPS <70). Kaplan–Meier survival analysis confirmed the existence of the three distinct risk classification groups identified by RPA, with the best outcome for class I (median, 33.6 month), the worst for class III (median, 11.7 month), and intermediate outcomes for classes II (Table 4 and Figure 2A). The global difference in OS between the classes, reflecting different survival categories, was highly statistically significant (p < 0.01). Table 4 shows the median OS and the survival rate at 0.5, 1, 3, and 5 years for each class. For instance, the survival rate of class I at 0.5, 1, 3, and 5 years were 100%, 88.9%, 66.7%, 61.9%, respectively. The outcomes of same trend were found in the validation set from CGGA (Figure 2B). Although, there was no significant difference of OS between class II and class III (p= 0.49), the median survival remained significantly different (class II 584, class III 438). When patients from the training and validation sets were combined, the three risk classification groups also remained significantly different (median OS of class I, class II and class III = 1009, 498 and 387 days Figure 2C). The p value between class I and class II by log-rank test was 0.0012 (class II and class III: p= 0.0246). According to the 2016 WHO glioblastomas classification [25]., we used RPA model specially in the IDH wild-type patients. Except for a slightly different number of patients in the nodes, the similar RPA model was observed in IDH wild-type cohort. Then, we validated the overall survival of patients in IDH wild-type cohort. The three distinct risk groups also had significance prognosis value (Figure 2D).

**Table 3:** Risk-group splits according to RPA

Class	No. of patients	MGMT	Age, y	KPS
**I**	21	Met	<58	Any
**II**	23	Met	≥58	Any
	11	Unm	<54	≥70
	26	Unm	>59	≥70
**III**	10	Unm	54-58	≥70
	12	Unm	Any	<70

RT: Radiotherapy; CT: Chemotherapy.

MGMT: MGMT promoter methylation; Met: Methylation; Unm: Unmethylation.

**Table 4:** Survival rate of TCGA patients in all Glasses

	Class I	Class II	Class III
**Median OS (month)**	33.6	15.9	11.7
**at 6-month (%)**	100	46.7	50
**at 1-year (%)**	88.9	77.1	44.4
**at 3-year (%)**	66.7	50	NR
**at 5-year (%)**	61.9	NR	NR

OS: Overall survival; NR: Not reached.

## DISCUSSION

Since its initial development in the early 1990s, the RPA classification system has been validated in multiple clinical trials [14]. In contrast with other measures of prognosis analysis, RPA allows enrollment of as many variables correlated with potential prognostic significance as desired. Undoubtedly, in RPA, the introduction of more significantly prognostic variables may help clarify some of the heterogeneity in patients and more realistically delineate patient prognosis. Malignant gliomas are known to comprise various heterogeneous populations. Previously, the molecular basis of heterogeneity in gliomas was confirmed. Therefore, comprehensive molecularly targeted treatment of GBM could play a critical role in the future. In addition, RPA avoided the bias inherent in subjective selection of evaluative variables for generating prognostic classifications.

We identified three different prognostic groups using readily available clinical variables and molecular parameters. There were only three variables identified in this classification schema: age, KPS and MGMT promoter methylation status. The most powerful variable was MGMT promoter methylation. Most prospective trials recognize the prognostic importance of KPS, and our analysis further categorized KPS as ≥70 or <70. Therefore, patients with different KPS could be accurately partitioned. Previously, some seminal Phase III studies used age cutoffs of 40 [15, 16] or 50 [17, 18] in randomization procedures or multivariate analyses. However, our results suggest that age band at 50-60 years may be the most important cut-points. KPS, age and MGMT promoter methylation were critical prognostic factors for OS in the univariate analysis (p < 0.05).

The RPA model produced in TCGA was excellent validated in CGGA and combined cohort. This RPA model could be more useful prognostic tool for patients with GBM. When we began to research this study, we wanted to use the more stable and more comprehensive databases to apply RPA algorithm. Because the most of the molecular information of the CGGA database was not publicly available for downloading. Then, the TCGA database contained a higher credible level. Other researchers could use this available database to verify our study results and to continue in-depth studies. Provided RPA for the CGGA cohort made the model become more complicated. Furthermore, the complex model was not conducive to the clinical application. In addition, we found that there were a few differences in age between CGGA and TCGA cohorts in the nodes when we used RPA to analyze the CGGA and TCGA cohorts respectively. It may be the results that Orientals and Westerns have difference risk of glioma. Thus, it need further research to investigate.

To our knowledge, RPA models have been generated and tested for brain tumors, like GBM (using only routine clinical factors: age, performance status, extent of resection, tumor site, and neurologic function) [8–10], anaplastic astrocytoma (using histology confirmed supratentorial GBM or astrocytomas with anaplastic or atypical foci, age, KPS, mental status, extent of surgery, and RT dose) [9], anaplastic oligodendroglioma (using age, tumor location, and only one molecular marker: deletion of chromosomes 1p and 19q) [12], and primary central nervous system lymphoma (using age and KPS) [13]. However, this is the first study to use various molecular parameters for RPA model construction to study prognosis in GBM. In some other RPA models some patients were treated with standard chemoradiation followed by adjuvant chemotherapy and some had either radiation or chemotherapy only. Currently, the chemoradiation and chemotherapy is the standard-of-care for GBM patients. It is of great value to generate models in patients with standard treatment instead of confirming the well-known conclusions that chemotherapy and radiation are prognostic factors for GBM patients. Thus, all enrolled patients were treated with standard chemoradiation, which this study can investigate more deeply the impact of molecular markers on patients with GBM.

The KPS describes a patient's functional status as a comprehensive 11-point scale correlating to percentage values ranging from 100% (no evidence of disease, no symptoms) to 0% (death). The KPS is an artificial construct which measures the ability to function. Important for survival is not the KPS percentage score, rather it is the disease state and co-morbidities, and the impact of these two items upon the patient's vitality [19]. The location of glioma growth, for example, did not involve the brain functional area and was easier to excise (frontal gliomas). These patients had higher score of KPS and higher opportunity to take total resection. These will undoubtedly increase the survival time of the patient. In this study, we used KPS to classify patients and guided clinical treatment programs. But, the further study can research the different survival and aberrant expression of molecular biomarkers in patients’ cohort with the similar tumor localization or the same extent of resection.

Corticosteroid (such as dexamethasone) was suggested to be immunosuppressant, as are radiotherapy and TMZ [20]. But a recent correlative study found that DEX treatment-induced immune suppression could interfere with clinical efficacy of standard therapy in recurrent glioblastoma [21]. In addition, Kenneth et. said use of corticosteroids early in the course of disease, during radiotherapy without or with chemotherapy, was an independent predictor of poor outcome in glioblastoma patients [22]. The EGFRvIII is expressed in approximately 20–30% of primary GBM and is not expressed on normal tissues, it is an effective target for immunotherapy [23]. These findings prompted us to enroll emerging molecular and clinical markers into RPA in further investigation work to allow this scoring system to be applied in routine clinical investigation to aid realistic patient prognosis.

Our analysis was limited by the retrospective nature of the original dataset and the lack of other central reviews, most notably its inherent associated biases. We also did not capture tumor size, which was previously shown to be prognostic for brain tumors [10]. Moreover, additional molecular abnormalities with potential prognostic significance beyond those examined herein have been discovered recently, and which of these can or should be routinely analyzed is an emerging area of investigation in neuro-oncology [24].

In conclusions, the RPA prognostic model presented herein was simple yet powerful. Although the model appears complex, we found that combining molecular and clinical data yielded three variables (MGMT promoter methylation, age, KPS) that powerfully predicted survival. Of these, MGMT promoter methylation was the most important factors influencing patients’ survival. To our knowledge, this was the first study to combine various molecular parameters and clinical factors into RPA to predict the prognosis of patients with GBM. We also included MGMT promoter methylation in our RPA model, which could give better suggestion to patients for their chemotherapy. This clinical RPA model will serve as the backbone for the future incorporation of molecular prognostic markers currently in development. Thus, our RPA model for GBM may aid in clinical prognosis evaluation.

## MATERIALS AND METHODS

### Patients and clinicopathological information

The corresponding clinical information (gender, age, Karnofsky Performance score), survival information and molecular biomarkers for 103 GBM patients were downloaded from The Cancer Genome Atlas (TCGA) database (http://cancergenome.nih.gov) as training set. Validation set containing one hundred and sixteen GBM patients were downloaded from CGGA database (http://www.cgga.org.cn). The clinical characteristics of patients from the two datasets were summarized in Table 1. We excluded patients with OS time of < 30 days, who might die due to other factors. All the patients (both in training dataset and validation dataset) were after standard treatment (operation and standard chemoradiation). The chemoradiation regimens were implemented for all patients as previously reported [25].

### Statistical analysis

Since RPA was developed in the early 1990s, it has been used to model predictors by building a pattern decision tree [26, 27]. RPA enables classification of patients into successively more homogeneous prognostic groups based on multiple input variables [7]. This recursive algorithm divides the data into two subgroups by searching through all possible prognostic variables, in terms of finding the division that is most prognostic for survival. Then RPA repeats the search to identify additional partitions within a previously identified subgroup. Each partition point is called as a node. The algorithm stops when the data have been divided into subgroups that are too small to divide further in a meaningful manner. The partitions and nodes build a tree, which allows for a clinically intuitive visual display of the factors examined and any potential interactions. The recursive PARTitioning package (RPART) was downloaded from https://cran.r-project.org/web/packages/rpart/index.html.

We constructed the tree using the following collected variables with potential prognostic significance: age (as a continuous variable), KPS at diagnosis, six molecular markers (IDH1/2, ATRX mutation status, mRNA expression level of TERT, 1p19q codeletion, TERT promoter mutation, and MGMT promoter methylation). Interestingly, the RPA originally generated six prognostic classes (Supplementary Figure 1). We combined some of the classes based on similar median survival.

The OS time was defined as time interval from histologic diagnosis of GBM to death or last follow-up. Univariate analysis was used to test the relationship between each prognostic factor and OS. Kaplan–Meier curves with log-rank test presented the final classification of prognostic groups.

## SUPPLEMENTARY FIGURE AND TABLE


