# Supplementary material for: Stratification according to recursive partitioning analysis predicts outcome in newly diagnosed glioblastomas
Source: Oncotarget. 2017 Apr 21;8(26):42974–82. doi: 10.18632/oncotarget.17322 (PMC5522120; doi:10.18632/oncotarget.17322)
Supplement: Supplementary file 1 [file oncotarget-08-42974-s001.pdf]

Stratification according to recursive partitioning analysis predicts outcome in newly diagnosed glioblastomas

SUPPLEMENTARY FIGURE AND TABLE

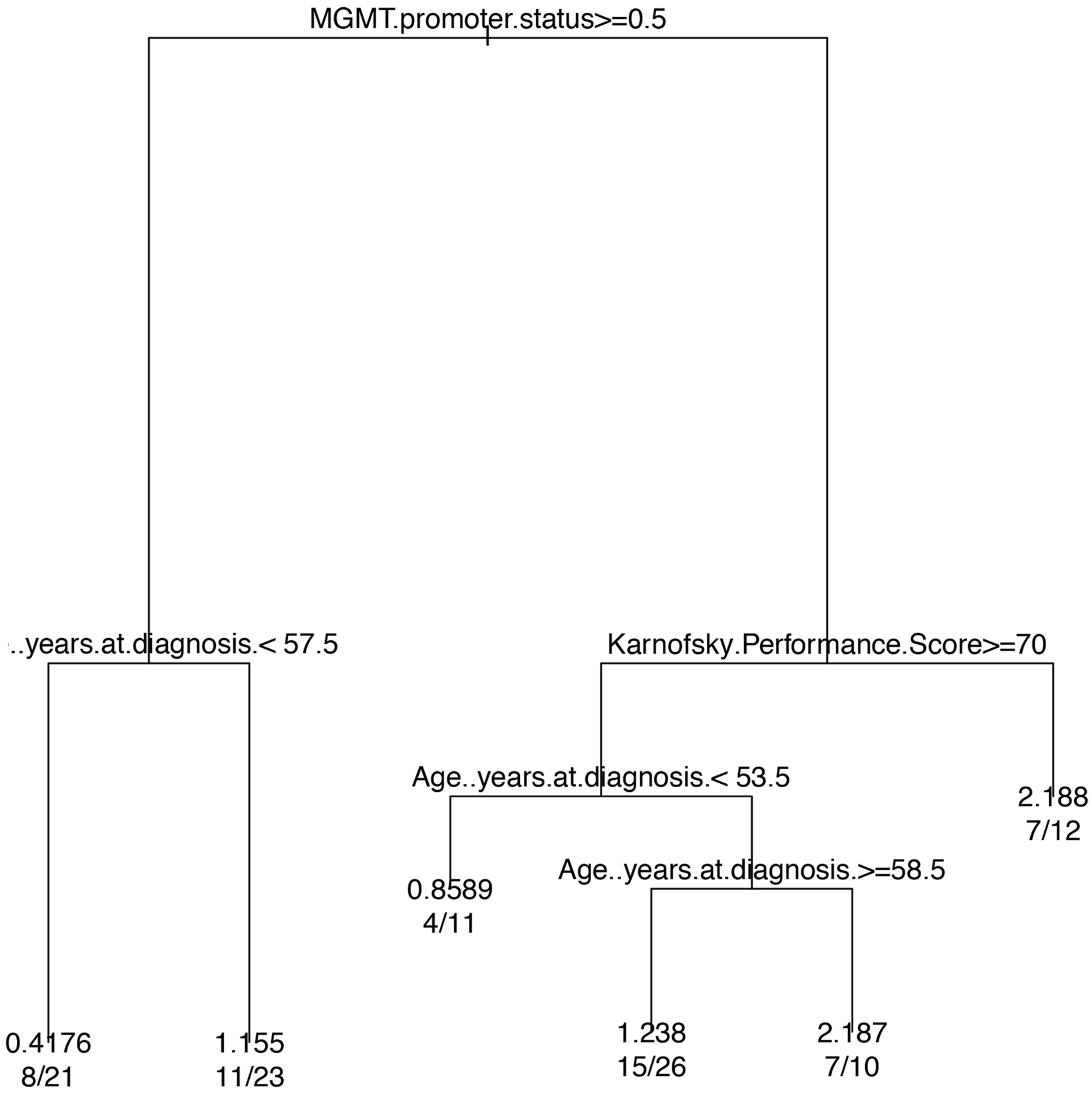

Supplementary Figure S1: The original RPA-generated tree with six leaves.

Supplementary Table 1: Univariate and multivariate analysis of 116 patients with GBM in CGGA

Variable	Total (n=116)					
	No. of patients	Median OS (days)	P value	HR	95%CI	P value
**Gender**						
**Men**	77	520	0.11			
**Women**	38	733				
**Age**						
**<49**	53	811	0.10	2.06	1.20-3.54	0.01
**≥49**	63	526				
**Preoperative KPS score**						
**<80**	50	372	<0.01	0.27	0.15-0.46	<0.01
**≥80**	66	811				
**ATRX mRNA expression**						
**High**	20	583	0.91			
**Low**	20	681				
**NA**	76					
**TERT mRNA expression**						
**High**	20	563	0.81			
**Low**	20	681				
**NA**	76					
**TERT promoter mutation**						
**mutation**	10		0.27			
**Wild type**	21	965				
**NA**	85	584				
**1p19q codeletion**						
**Codeletion**	1	372	0.19			0.53
**Non-codeletion**	115	657				
**IDH mutation**						
**Mutation**	18	970	0.48			
**Wild type**	98	657				
**MGMT promoter methylation**						
**Methylation**	43	809	0.30	0.59	0.35-1.01	0.05
**Unmethylation**	73	369				

OS: overall survival; HR: Hazard ratio
